# Dementia Improvement after Plasma Exchange for Familial Hypercholesterolemia

**DOI:** 10.1155/2016/6121878

**Published:** 2016-09-08

**Authors:** Allen J. Orehek

**Affiliations:** Dementia Prevention Center, 1 Montage Mountain Road, Moosic, PA 18507, USA

## Abstract

Worldwide dementia related memory issues affect a great number of patients and families. In this case, a “senior moment” was noted at age fifty and issues with memory and mind progressed resulting in early retirement from work. The patient described here was given a diagnosis of “Pre-Alzheimer's disease” and presented for further accurate evaluation, diagnosis, and management. The medical management resulted in an improvement in the patients memory and cognitive ability.

## 1. Introduction

Dementia and cognitive function loss from a variety of etiology are common [1]. Familial hypercholesterolemia related elevations in low-density lipoprotein (LDL) are often challenging to treat [2]. This case describes some of the changes in cognitive function that were observed over time.

## 2. Case

At age 15 she had a syncope and collapse where no diagnosis was made. At age 50 the family noticed the first problem with memory. The patient purchased and picked up a birthday cake from local bakery, yet later the same evening she asked who brought the cake, apparently unaware of the day's events. Through her fifties and sixties a steady variety of cognitive difficulties were noted by family and patient. Being an avid reader she became frustrated with inability to remember character development. On her own as a memory tool she started to write character's attributes into front covers. The patient began to have difficulty with ordering meals from menu selections. She was unable to properly purchase a list at the grocery store, often arriving home with items missing. Age 62 she was treated with lumpectomy and radiation for breast cancer. Around that time she was placed on medication for elevation of LDL due to familial hypercholesterolemia. At age 67 she began to notice difficulty with remembering product codes related to her job. Two years later when the difficulty with work progressed due to issues with her memory she retired. Her medical team explained that she had symptoms of Alzheimer's disease. At age 71 she presented for evaluation of issues with memory and mind. Medications included rosuvastatin 40 mg/d, citalopram 10 mg/d, aspirin 81 mg/d, and ezetimibe 10 mg/d. Presenting LDL on medications was 220 mg/dL. There were no other significant hospitalizations or medical diagnosis aside from the ones mentioned. She did not use tobacco in her life. Family members were also affected with hypercholesterolemia. The father passed away in his fifties from a sudden cardiac event. The children are known to have LDL levels over 500 mg/dL. Younger sister after loss of driver's license had completion of neuropsychiatric testing and was given a diagnosis of cognitive loss related to “Pre-Alzheimer's disease.”

Physical exam provided well developed well nourished elderly female. BP was 120/74 mm/Hg. Eye exam revealed corneal arcus without the presence of xanthelasmas. Auscultation revealed a 3/6 mid-systolic cardiac murmur and bilateral carotid bruits or transmitted cardiac murmur sounds, flattening and asymmetry of buccinator movement with preserved function (cranial nerve VII), enlargement of thyroid, possible nodule, and metacarpophalangeal joint tendon xanthomas.

Medical evaluation revealed brain MRI findings of enlargement of bilateral ventricles, loss of neuronal mass, enlargement of cerebral cortex sulcus and shrinkage of gyri, and periventricular and posterior FLAIR signal changes, along with numerous isolated signal changes. A 24-hour Holter monitor recorded rare nonsustained supraventricular tachycardia. 2-dimensional echo revealed the following: 26 mm aortic root with calcifications, sclerotic trileaflet aortic valve, calcified thickened mitral valve with mild insufficiency, left atrium diameter 37 mm, and ejection fraction estimated at 60 percent. Noninvasive myocardial perfusion imaging testing was completed and reported as a homogenous distribution with no perfusion defects. Doppler ultrasound revealed 20–49% bilateral stenosis of carotid arteries. A thyroid ultrasound found homogenous enlargement of the gland without any discrete nodule noted. Apolipoprotein B was 115 mg/dL. ANA was negative. Homocystine was 8.99 *μ*mol/L, and uric acid was 3.7 mg/dL. For genetic mutation in factor 5 Leiden with a R506Q mutation, there was no point mutation (G20210A) in factor 2 genetic testing, a homozygous MTHFR C677T mutation was found, and APO E genotyping resulted in E3/E4. The following additional genetic testing was completed in this case: PC3K9 defects noted as c.1026A>G p.Gin342Gin (synonymous mutation homozygous) c.1380A>G P.Va460Val (synonymous mutation homozygous), c.1420G>A pVa474lle (missense mutation homozygous), c.2009G>A p.Gly670Glu (missense mutation homozygous), and c.207+15A>G, NA, intronic mutation homozygous, and otherwise normal renal, thyroid, and blood chemistry. Independent neuropsychological testing and interpretation diagnosed memory loss and vascular dementia. The patient had weakness in verbal memory, as well as visual and design memory and verbally mediated executive functioning. Testing also added adjustment disorder with mixed anxiety and depressed mood.

After updating all patient data and gathering information a large decision tree existed with multiple management options (e.g., factor V, homocysteine, and carotid stenosis). The difficulty in medical management of familial hypercholesterolemia and familial combined hyperlipidemia has been previously described [3]. Initial patient management included a drug-free period and follow-up lab data. After four weeks there was a significant elevation in LDL. The patients untreated LDL was 351 mg/dL. Maximum lifestyle, diet, and medications (rosuvastatin 40 mg/d and ezetimibe 10 mg/d) were not effective in achieving a lower LDL. After ruling out many of the easily identified reasons for cognitive decline and accepting that more obscure diagnosis could be possible the patient wanted to complete a period of LDL mitigation and observation. The patient and family made a decision to treat the elevated LDL not responsive to current management with selective lipoprotein apheresis [4, 5]. Selective LDL apheresis equipment was not geographically available at the apheresis clinic and the patient was offered plasma exchange as best available geographic option. The patient started plasma exchange sessions shortly after. Therapeutic plasma exchanges were carried out with an automated blood cell separator. Peripheral intravenous access would be obtained and 2451 mL of 5% albumin 180 mL of normal saline would be exchanged during therapeutic plasmapheresis (a goal of 35 mL/kg of plasma was removed and the geographically available facility would use normal saline in additional volume replacement). There were never any reported or perceived complications or side effects of any of the treatment sessions. After two years of plasma exchange with 5% albumin on an every-3-week schedule the patients LDL range was 46–114 mg/dL. Cognitive improvement was gradual but after years specific events were described. The patient was able to go to the store with a list of groceries and bring them all home. Interaction with a restaurant menu no longer became a challenge and family notices this as most striking compared to the past where the interaction of selecting from a menu and communicating that was difficult. The patient explained that she had less problems with her books, being able to follow a detective story from the beginning to the end without taking extra notes. At twenty seven months a follow-up carotid artery noninvasive color flow duplex vascular ultrasound study was completed by the patient. Comparisons of the velocities show some improvements. Compared to the initial study there were some changes in the velocities: proximal common carotid artery initial study, right 123 cm/s and left 147 cm/s, with follow-up study, right 104 cm/s and left 118 cm/s, and the mid internal carotid artery initial study, right 111 cm/s and left 99.6 cm/s, with follow-up study, right 94.6 cm/s and left 75.6 cm/s.

## 3. Discussion

The patient's initial presentation is a common one where memory issues are noted by patient and family, yet the issues are challenging to measure, grade, score, or judge to any degree of certainty. The finding of LDL elevations is not uncommon. After medical management, patient choice apheresis was a safe and effective treatment [6]. Access to repeat neuropsychiatric testing would be helpful but geographic and insurance issues limited patient access. There are guidelines for the use and indications of lipoprotein apheresis along with debate on the same guidelines [7]. Guidelines for use of lipoprotein apheresis that do not include any indications of data obtained from levels of Apo B could represent an area of interest. Limitations: in this case there may have been an added benefit of more frequent sessions; however the patient's balance of lifestyle and access did not afford more frequent sessions. Using the interactions of daily affairs as observed by the patient or family as a marker of changes in cognitive ability is challenging (e.g., success with grocery lists and interaction with menus), yet in this case there is enough improvement for the family and patient to provide extremely strong feedback of improvement that it deserves description. There could have been other lifestyle or physiologic reasons that contributed to the improvement in cogitative ability that remain unidentified in part or whole, for example, effects of plasma exchange on factor 5 genetic mutation, homocystine, fibrinogen, coagulation, or endothelium vasodilatation [8]. Therapeutic plasma exchange inherently provides significant nonlipid anti-inflammatory effect useful across a plethora of disorders [9, 10]. The removal of proinflammatory factors (including complement, coagulation factors, and cytokines, and endothelial adhesion molecules) may provide additional benefits beyond the LDL reduction [11]. With the removal of intravascular plasma proteins other authors have shown variable results and response to therapeutic plasma exchange [12]. While it remains elusive to place an exact time on the improvement as cognitive ability has many different associated factors, yet family and patient after two years of treatment reported a significant improvement in her day to day function. She was again able to read books and literature, she was able to properly plan and handle family events, and daughter present at each visit reported remarkable ability of the patients recall on a daily basis. Many more attributes of the unique individual are unable to be accurately evaluated that may be perceived as significant cognitive improvement. (e.g., self-confidence, mood, compassion, curiosity, empathy, concentration, enjoying life events, and creativity). During a visit at three years of treatment the patient reported that she was continually able to read detective books without the need to place notes and reminders of the characters in the front cover. These attributes may also contribute to the cognitive ability of the brain to function at an improved degree. Data such as level of LDL, neuropsychiatric scores, and brain atrophy are better suited to be measured, graded, and separated into scientific categories. Many have described the expected course of a patient diagnosed with Alzheimer's, yet there remain fewer described cases of improvement after that diagnosis [13, 14]. Others have described reversal of dementia [15–17]. This case may deserve wider attention as this is a common patient population. Similar patients have been described who are left with inadequate therapy without access to lipopheresis [18]. Prevention of dementia and improvement in cognitive function remain obscure and difficult as there are no good tests of what makes up the attributes of a unique individual.

## References

[1] Harvey R. J., Skelton-Robinson M., Rossor M. N. (2003). The prevalence and causes of dementia in people under the age of 65 years. *Journal of Neurology, Neurosurgery & Psychiatry*.

[2] Thompson G. R. (2003). LDL apheresis. *Atherosclerosis*.

[3] Williams R. R., Hunt S. C., Schumacher M. C. (1993). Diagnosing heterozygous familial hypercholesterolemia using new practical criteria validated by molecular genetics. *The American Journal of Cardiology*.

[4] Vella A., Pineda A. A., O'Brien T. (2001). Low-density lipoprotein apheresis for the treatment of refractory hyperlipidemia. *Mayo Clinic Proceedings*.

[5] Beigel R., Beigel Y. (2009). Homozygous familial hypercholesterolemia: long term clinical course and plasma exchange therapy for two individual patients and review of the literature. *Journal of Clinical Apheresis*.

[6] Thompson G. R. (2008). Recommendations for the use of LDL apheresis. *Atherosclerosis*.

[7] Austin M. A., Brunzell J. D., Fitch W. L., Krauss R. M. (1990). Inheritance of low density lipoprotein subclass patterns in familial combined hyperlipidemia. *Arteriosclerosis, Thrombosis, and Vascular Biology*.

[8] Tamai O., Matsuoka H., Itabe H., Wada Y., Kohno K., Imaizumi T. (1997). Single LDL apheresis improves endothelium-dependent vasodilatation in hypercholesterolemic humans. *Circulation*.

[9] Mokrzycki M. H., Kaplan A. A. (1994). Therapeutic plasma exchange: complications and management. *American Journal of Kidney Diseases*.

[10] Jayne D. R. W., Gaskin G., Rasmussen N. (2007). Randomized trial of plasma exchange or high-dosage methylprednisolone as adjunctive therapy for severe renal vasculitis. *Journal of the American Society of Nephrology*.

[11] Tesař V., Jelínková E., Mašek Z. (1998). Influence of plasma exchange on serum levels of cytokines and adhesion molecules in ANCA-positive renal vasculitis. *Blood Purification*.

[12] Keegan M., König F., McClelland R. (2005). Relation between humoral pathological changes in multiple sclerosis and response to therapeutic plasma exchange. *The Lancet*.

[13] Amieva H., Jacqmin-Gadda H., Orgogozo J.-M. (2005). The 9 year cognitive decline before dementia of the Alzheimer type: a prospective population-based study. *Brain*.

[14] Leoutsakos J.-M. S., Forrester S. N., Corcoran C. D. (2015). Latent classes of course in Alzheimer's disease and predictors: the Cache County Dementia Progression Study. *International Journal of Geriatric Psychiatry*.

[15] Gunstad J., Strain G., Devlin M. J. (2011). Improved memory function 12 weeks after bariatric surgery. *Surgery for Obesity and Related Diseases*.

[16] Walstra G. J. M., Teunisse S., Van Gool W. A., Van Crevel H. (1997). Reversible dementia in elderly patients referred to a memory clinic. *Journal of Neurology*.

[17] Ansari I., Grossberg G. T. (2015). Syphilis a reversible cause of dementia. *Journal of Gerontology & Geriatric Research*.

[18] Brown W. V., Brook R., Hemphill L. C., Moriarty P. M. (2012). The use of lipopheresis in the practice of clinical lipidology. *Journal of Clinical Lipidology*.

